# A genome‐wide association study of morphometric traits in dromedaries

**DOI:** 10.1002/vms3.1151

**Published:** 2023-05-04

**Authors:** Morteza Bitaraf sani, Omid Karimi, Pamela Anna Burger, Arash Javanmard, Zahra Roudbari, Mokhtar Mohajer, Nader Asadzadeh, Javad Zareh Harofteh, Ali Kazemi, Ali Shafei Naderi

**Affiliations:** ^1^ Animal Science Research Department Yazd Agricultural and Natural Resources Research and Education Center Agricultural Research Education & Extension Organization (AREEO) Yazd Iran; ^2^ Department of Animal Viral Diseases Research Razi Vaccine and Serum Research Institute Agricultural Research Education and Extension Organization Karaj Iran; ^3^ Research Institute of Wildlife Ecology Vetmeduni Vienna Vienna Austria; ^4^ Department of Animal Science Faculty of Agriculture University of Tabriz Tabriz Iran; ^5^ Department of Animal Science Faculty of Agriculture University of Jiroft Jiroft Iran; ^6^ Animal Science Research Institute of Iran Agricultural Research Education and Extension Organization (AREEO) Karaj Iran; ^7^ Animal Breeding Canter of Iran Karaj Iran

**Keywords:** *Camelus dromedarius*, candidate genes, genotyping by sequencing, single nucleotide polymorphism (SNP)

## Abstract

**Background:**

Investigating genomic regions associated with morphometric traits in camels is valuable, because it allows a better understanding of adaptive and productive features to implement a sustainable management and a customised breeding program for dromedaries.

**Objectives:**

With a genome‐wide association study (GWAS) including 96 Iranian dromedaries phenotyped for 12 morphometric traits and genotyped‐by‐sequencing (GBS) with 14,522 SNPs, we aimed at identifying associated candidate genes.

**Methods:**

The association between SNPs and morphometric traits was investigated using a linear mixed model with principal component analysis (PCA) and kinship matrix.

**Results:**

With this approach, we detected 59 SNPs located in 37 candidate genes potentially associated to morphometric traits in dromedaries. The top associated SNPs were related to pin width, whither to pin length, height at whither, muzzle girth, and tail length. Interestingly, the results highlight the association between whither height, muzzle circumference, tail length, whither to pin length. The identified candidate genes were associated with growth, body size, and immune system in other species.

**Conclusions:**

We identified three key hub genes in the gene network analysis including *ACTB, SOCS1* and *ARFGEF1*. In the central position of gene network, ACTB was detected as the most important gene related to muscle function. With this initial GWAS using GBS on dromedary camels for morphometric traits, we show that this SNP panel can be effective for genetic evaluation of growth in dromedaries. However, we suggest a higher‐density SNP array may greatly improve the reliability of the results.

## Graphical abstract

We detected 59 SNPs located in 37 candidate genes potentially associated to morphometric traits in dromedaries. We identified three key hub genes in the gene network analysis including *ACTB*, *SOCS1* and *ARFGEF1*. In the central position of gene network, *ACTB* was detected as the most important gene related to muscle function.

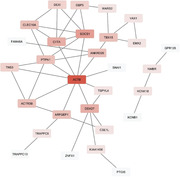

## 1 INTRODUCTION

Since ancient times, domestic even‐toed ungulates have played pivotal roles for man, being exploited for meat and milk, for fibre production, as beasts of burden for transport in agricultural/ rural oriented community; they were even worshipped (Barazandeh et al., 2019). These animals have served for traditional technologies since the very early era of domestication (Barazandeh et al., 2016). The Bactrian camel was domesticated, probably in northern of Iran, northeast Afghanistan or southwestern Turkmenistan (Mohammadabadi et al., 2021). Camels have been producing meat, milk and wool in desert conditions for thousands of years. Among the 35 million camels in the world, 95% are one‐humped camels (Hashim et al., 2015). The number of Iranian camels is 152,346, which are being bred in the southern and central deserts of Iran (FAO, 2021). The most breeding units are in Yazd, Kerman, Semnan and Sistan, and Baluchistan provinces. Camel meat in Iran, with the production of four and a half thousand tons, includes 1% of the country's total red meat production (Khodai, 2004). This is even though the amount of meat and milk production in Iran is very low in general, and the declining trend of camel population in the country in recent years is worrying. To foster camel breeding for sustainable animal protein production from desert climate resources, attention to genetic improvement is of particular importance. Successful breeding programs in camels face basic problems such as lack of sufficient phenotype records, missing pedigree, small herd size and lack of kinship relationships. Furthermore, the morphometric recording can be notoriously difficult and prone to injuries caused by dromedaries not accustomed to measuring procedures. Thus, no systematic breeding program has been carried out in Iran, so far. In camel breeding programs, survival traits, reproductive traits (pregnancy rate, calving interval and age at first calving) and production traits including calves’ and camel's weights should be considered as breeding goals (Vatankhah et al., 2019). Vatankhah et al. (2019) reported the economic value of calving interval to be −13.19$/month. To preserve and improve the genetic stock of camels and increase the productivity of meat production, it seems necessary to use new genomic technologies.

Due to the long generation interval of camels, it takes many years to achieve genetic improvement using traditional breeding, and it is practically impossible to achieve this with the usual methods. On the other hand, the cost of breeding in the traditional way and based on progeny testing is very high compared to modern genomic methods. By using modern breeding methods, including the use of customised low‐density SNP arrays, the speed of genetic progress can be significantly increased by using selection at a young age. The SNPs and genes associated with morphometric traits, especially height at withers, body length, hip width, breast circumference and scrotal circumference in cattle have been presented with several GWAS studies (Bolormaa et al., 2011; Cole et al., 2011; Terakado et al., 2018). The heritability of these traits is mostly reported to be moderate to high (An et al., 2019; Munim et al., 2013; Zhang et al., 2017). Despite the fact that the genomic structure of cattle morphometric traits is highly polygenic, there are many similarities with other livestock species, humans and mammals in general (Bouwman et al., 2018; Pryce et al., 2011). The identified regions were mainly involved in biological functions such as regulation of embryonic development, skeletal development, regulation of cell cycle or cell division, homeostasis and lipid metabolism (Bouwman et al., 2018; Pryce et al., 2011; Setoguchi et al., 2011). More interestingly, different studies have identified significant overlaps in genomic architecture and genomic relationships between morphometric traits and cattle performance characteristics, including body weight, carcass traits, feed consumption, reproduction, and health, and in other words, phenotypic correlation between morphometric traits and animal performance such as body weight, milk production is similar in different breeds (An et al., 2019; Bilal et al., 2016; Lukuyu et al., 2016; McKenna et al., 2010; Misganaw et al., 2013; Pryce et al., 2011; Thomas et al., 2021). In addition, models have been developed to predict body weight using chest girth, body length, or height at whither (Lukuyu et al., 2016; Thomas et al., 2021), which suggests that morphometric traits are important predictors of animal performance in African domestic livestock herds, which have a weak recording system. Addressing the principles of selection, several studies showed that population morphometric diversity is the result of selection for adaptive and sociocultural interests in small livestock production in Africa (Kabi et al., 2015; Yougbaré et al., 2020; Yougbaré et al., 2021).

Therefore, investigating the genomic regions associated with morphometric traits in camels is valuable, because it allows a better understanding of adaptive and productive features. In addition, it opens the prospect of effective use of morphometric traits in recording the basic phenotype for any potential community‐based breeding program (Ouédraogo et al., 2021; Ouédraogo et al., 2021). With a GWAS approach, we aimed at identifying candidate genes potentially associated to morphometric traits to develop the basis for a sustainable breeding program for dromedaries in Iran.

**SIMPLE SUMMARY**

With a genome‐wide association study (GWAS) including 96 Iranian dromedaries phenotyped for 12 morphometric traits and genotyped‐by‐sequencing (GBS) with 14,522 Single Nucleotide Polymorphisms (SNPs), we aimed at identifying associated candidate genes. We detected 59 SNPs located in 37 candidate genes potentially associated to morphometric traits in dromedaries. The top associated SNPs were related to pin width, whither to pin length, height at whither, muzzle girth, and tail length. We identified three key hub genes in the gene network analysis including Actin Beta *(ACTB*), Suppressor Of Cytokine Signalling 1 *(SOCS1*) and ADP Ribosylation Factor Guanine Nucleotide Exchange Factor 1 *(ARFGEF1*). In the central position of gene network, *ACTB* was detected as the most important gene related to muscle function.

## 2 MATERIALS AND METHODS

### 2.1 Phenotypic measured traits

**FIGURE 1:**
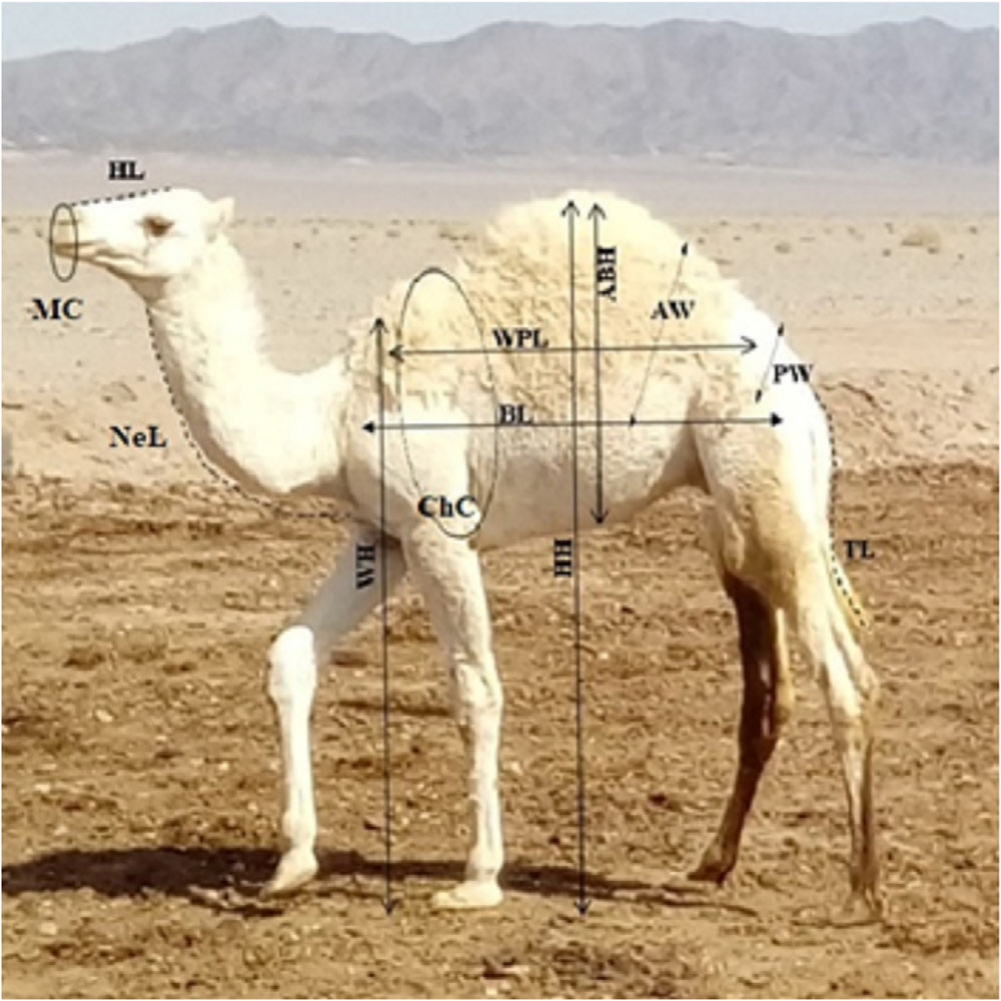
Schematics of 12 morphometric traits in dromedaries.

The number of 255 records of 12 morphometric traits were collected from 96 Iranian male camels during birth to 6 months old (every 3 months) in 2018. Morphometric traits included head length (HL), muzzle circumference (MC), neck length (NeL), chest circumference (ChC), whither height (WH), hump height (HH), whither to pin length (WPL), body length (BL), tail length (TL), pin width (PW), abdominal width (AW) and abdominal hump height (ABH). Schematics of these physical parameters are shown in Figure 1.

Head length (HL), muzzle circumference (MC), neck length (NeL), chest circumference (ChC), whither height (WH), hump height (HH), whither to pin length (WPL), body length (BL), tail length (TL), pin width (PW), abdominal width (AW), and abdominal hump height (ABH).

### 2.2 Ethical statement for blood collection in the dromedaries

All procedures were performed in strict accordance with the guidelines and regulations proposed by the Animal Science Research Institute of Iran. All the animal experiments were approved by ethics committee of the Animal Science Research Institute of Iran under the number ASRI‐34−64‐1357−005‐970,180. Blood samples were collected during qualified veterinary treatment within the framework of governmental programs aimed at the animal identification, monitoring of health, and parentage confirmation of the dromedary populations included in our study. No other kind of tissue was used in this study.

### 2.3 GBS technique and Bioinformatic analysis

The 96 Blood samples were gathered from jugular vein using EDTA tubes. The genomic DNA was extracted using the modified salting‐out method and quantified using spectrophotometry and checked for quality on a 1% agarose gel (Javanrouh et al., 2006). The samples were genotyped using GBS, which involves several steps; first, cutting the DNA and producing fragments using two restriction enzymes (*EcoR1* and *HinF1*); second, connecting the adapter to the cut fragments and removing very small fragments; and finally amplification of fragments using DNA polymerase. Paired‐end (150 bp) next‐generation sequencing (10 X) on the Illumina HiSeq 2000 platform was performed by Bayan Gene Pars Company. The produced sequences were mapped with the assembled genome of the camel (assembly accession: GCA_000803125.3) using the BWA‐MEM algorithm and software (BWA) (Li & Durbin, 2010). PCR Duplicates were detected using the Picard tool and disregarded in downstream analyses both by GATK (Ouédraogo et al., 2021) and SAMtools (Pembleton et al., 2013). SNPs were called across the GBS data using GATK and with a filter for a minor allele frequency (MAF < 0.01). The association between SNPs and morphometric traits was investigated using a linear mixed model with PCA and kinship matrix by TASSEL software (Bradbury et al., 2007). Geographical region and calf age were included as fixed and covariate effects in the model, respectively. The MLM_PCA+K statistical model was applied as follows:

y=αX+βP+uZ+e,

where *y* was the phenotype value; α was the vector of SNP effects; *β* was vector of population structure effects based on PCA; *u* was vector of kinship background effects; *e* was vector of residual effects; *X*, *P*, *Z* were incidence matrix relating the individuals to fixed marker effects α, fixed principal component (PC) effects *β*, random group effects *u*, respectively.

The suggestive significant Bonferroni *p*‐value thresholds were set (−log *p*‐value > 4) using the GEC software tool (Li et al., 2012). The associated SNPs (−Log *p*‐value > 4) were traced in NCBI and the candidate genes were detected by blasting to the dromedary camel's reference genome (GCA_000803125.3). We considered genes associated with the respective SNPs, if they were located either within the exon/ intron of a gene or within a flanking region of 50 kb up‐ and downstream of the SNP. The Search Tool for the Retrieval of Interacting Genes (STRING) version 11.5 (https://string‐db.org) was used to explore protein‐protein interaction (PPI) networks (Shannon et al., 2003). A combined interaction score of > 0.4 was considered significant. The PPI networks were visualised using Cytoscape software (http://www.cytoscape.org) (Szklarczyk et al., 2015). The *p*‐value ≤ 0.05 was considered to indicate a statistically significant difference. The topology scores of the nodes in the PPI network were estimated based on closeness centrality, betweenness centrality and degree centrality

## 3 RESULTS

### 3.1 Morphometric measurements

**FIGURE 2:**
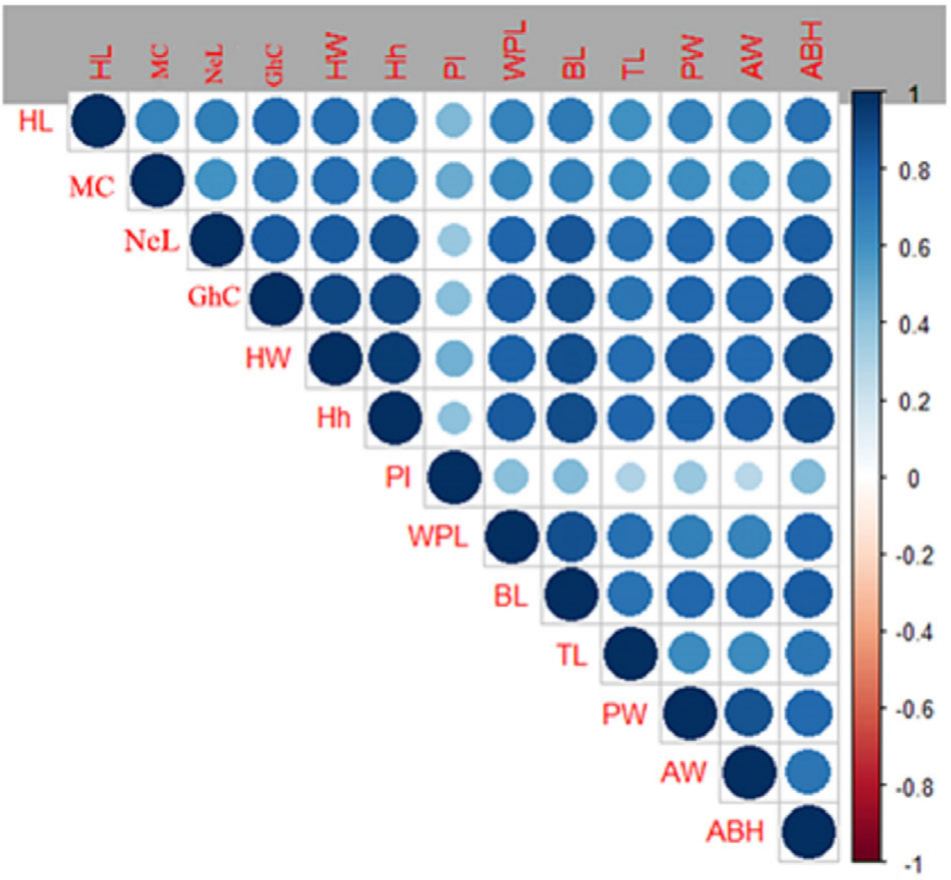
The correlation coefficients of morphometric records in dromedaries.

Descriptive statistics of morphometric traits are given in Table 1, while the corresponding correlation coefficients are visualised in Figure 2. Coefficient of variation (CV) of morphometric records ranged 0.10 to 0.27. There was high correlation among morphometric records so that most of them were significantly correlated.

**TABLE 1:** Mean, standard error (SE) and coefficient of variation (CV) of 255 morphometric records in dromedaries.

morphometric characters (cm)	Mean ± SE	CV
HL	33.27 ± 0.32	0.13
MG	30.73 ± 0.29	0.13
NeL	60.10 ± 0.93	0.19
GhC	113.73 ± 1.77	0.19
WH	128.02 ± 1.10	0.10
HH	134.28 ± 1.30	0.12
WPL	47.43 ± 1.03	0.27
BL	84.75 ± 1.39	0.20
TL	37.62 ± 0.40	0.13
PW	26.48 ± 0.41	0.19
PL	77.2 ± 0.33	0.16
AW	31.80 ± 0.36	0.24
ABH	49.83 ± 0.80	0.20

### 3.2 Associated SNPs and candidate genes

**FIGURE 3:**
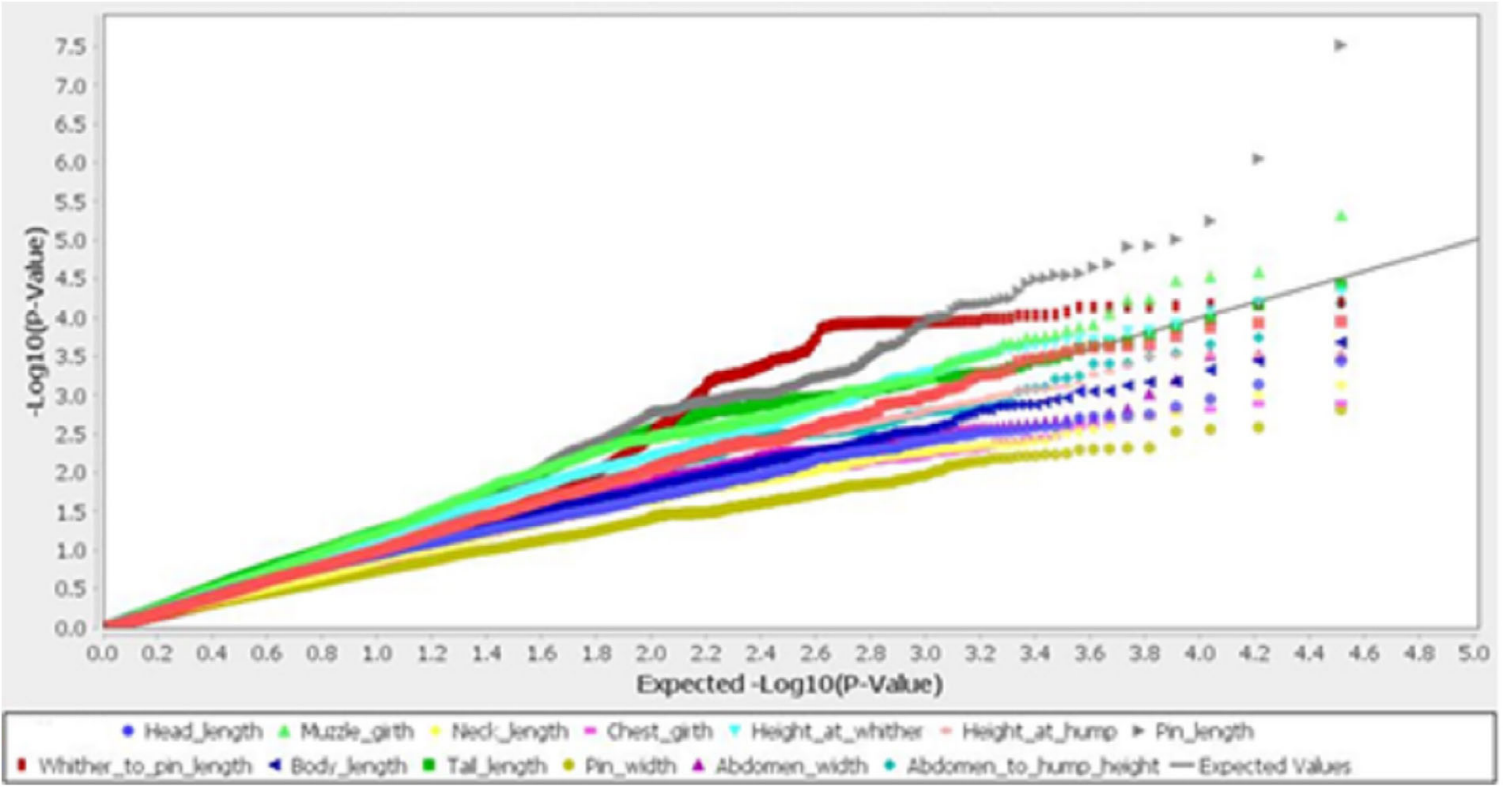
Q‐Q plot displays GWAS results from TASSEL for morphometric traits in dromedaries.

There were no determined clusters among geographical regions. Only 1.6% and 1.4% of the genetic variance was illustrated with PC1 and PC2, respectively, which demonstrated that calves are relatively homogeneous. A genome‐wide association study (GWAS) was performed using 14,522 markers and detected 59 SNPs associated with morphometric traits (Figure 3 and Table 2) at a cut‐off −log *p*‐value > 4. The most associated SNPs were related to pin width and whither to pin length (Table 2). Height at withers was significantly associated with three SNPs located on chromosomes 9 and 18. The seven SNPs, located on chromosome 11, 25, 19 and X, were associated with muzzle girth and two SNPs were associated with tail length. *ZNF326, GBP5, WARS2, TBX15, DEXI, CIITA, TSPYL4, EMX2, PRSS47, PTPN1, TRAPPC13, MAGEA2, KCNK18, VAX1, CLEC16, SOCS1, ADGRG6, NMBR, ACTR3B, ANKRD20A12P, PTGIS, POFUT2, TRAPPC9, ANKRD26, DDX27, ARFGEF1, CSE1L, SUPT20H, SNAI1, TNS3, TRMT9B, F5, SBSPON, ZNFX1* and *KCNB1* are considered as candidate genes related to morphometric traits.

**TABLE 2:** Genome and chromosome‐wide significant SNPs and potential candidate genes associated with morphometric traits in dromedaries.

Morphometric trait	SNP	Chr	Position	–Log (*p*‐value)	Candidate gene
Height at whither	Chr9_544346	9	544346	4.32	*ZNF326, GBP5*
	Chr9_22775986	9	22775986	4.20	*WARS2, TBX15, ZNF326*
	Chr18_29939174	18	29939174	4.08	*DEXI, CIITA, TSPYL4*
Muzzle circumference	Chr11_72352019	11	72352019	5.31	*EMX2*
	Chr25_263414	25	263414	4.58	
	Chr25_263446	25	263446	4.52	
	Chr25_263438	25	263438	4.47	
	Chr25_263458	25	263458	4.23	*PRSS47*
	Chr19_9739222	19	9739222	4.22	*PTPN1*
	Chrx_62115102	X	62115102	4.05	*TRAPPC13, MAGEA2*
Pin width	Chr11_72011327	11	72011327	7.51	*KCNK18, VAX1*
	Chr18_29995000	18	29995000	6.05	*DEXI, CLEC16A*
	Chr14_31105116	14	31105116	5.24	
	Chr18_29996796	18	29996796	5.00	*DEXI*
	Chr11_72056947	11	72056947	4.91	*KCNK18*
	Chr16_20502	16	20502	4.69	
	Chr18_30121089	18	30121089	4.64	*CLEC16A, SOCS1*
	Chr18_29983318	18	29983318	4.57	*TSPYL4*
	Chrx_46140989	X	46140989	4.54	
	Chrx_46141007	X	46141007	4.54	
	Chr8_60334433	8	60334433	4.51	*ADGRG6, NMBR*
	Chrx_46295476	X	46295476	4.49	
	Chr7_82469691	7	82469691	4.44	*ACTR3B*
	Chr17_21014	17	21014	4.34	
	Chr6_35301	6	35301	4.25	*ANKRD20A12P*
	Chr19_10394699	19	10394699	4.24	*PTGIS*
	Chr14_31079313	14	31079313	4.22	
	Chr25_71430	25	71430	4.19	
	Chr14_31079246	14	31079246	4.18	*TRAPPC9, POFUT2*
	Chr14_31079211	14	31079211	4.17	*TRAPPC9, POFUT2*
	Chr17_502	17	502	4.16	
	Chr25_71437	25	71437	4.16	
	Chr35_5668518	35	5668518	4.16	*ANKRD26*
	Chr14_30823324	14	30823324	4.14	*DDX27, ARFGEF1, CSE1L*
	Chr19_10802692	19	10802692	4.09	*ARFGEF1, CSE1L*
	Chr14_31091109	14	31091109	4.01	*SUPT20H*
	Chr14_31091156	14	31091156	4.01	
	Chr14_31091096	14	31091096	4.01	
Tail length	Chr19_10187187	19	10187187	4.42	*SNAI1*
	Chr25_263945	25	263945	4.16	
Whither to pin length	Chr14_31102269	14	31102269	4.20	
	Chr25_64593	25	64593	4.18	
	Chr36_1239778	36	1239778	4.17	*TNS3*
	Chr26_11514616	26	11514616	4.15	*TRMT9B*
	Chr19_9781745	19	9781745	4.14	*PTPN1*
	Chr19_10446024	19	10446024	4.14	*PTGIS*
	Chr8_59032939	8	59032939	4.13	
	Chrx_6887601	X	6887601	4.13	
	Chrx_6887611	X	6887611	4.13	
	Chr7_15583	7	15583	4.07	
	Chrx_6606858	X	6606858	4.04	
	Chr14_30937331	14	30937331	4.03	
	Chr14_30937412	14	30937412	4.03	
	Chr21_12524207	21	12524207	4.02	*F5*
	Chr11_71979910	11	71979910	4.02	*VAX1*
	Chr19_10589993	19	10589993	4.00	*SBSPON* *ZNFX1, KCNB1*

### 3.3 Protein‐protein interaction (PPI) network construction and hub gene identification

**FIGURE 4:**
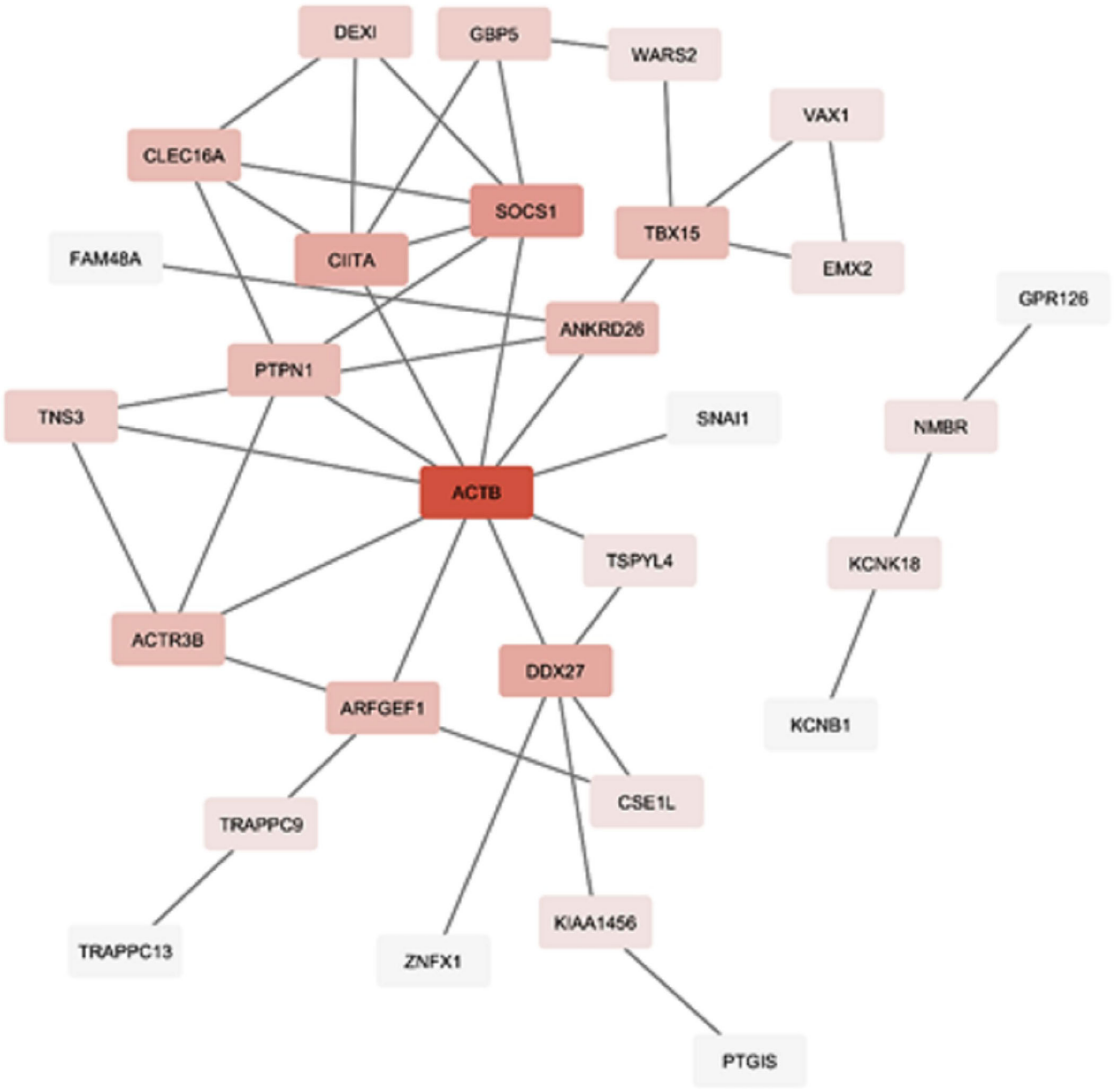
Network of candidate genes related to morphometric traits in dromedaries.

A PPI network was established to investigate the interaction between the common DEGs using the STRING database and Cytoscape software (Figure 4). Further analysis revealed that these candidate genes were linked to nine ontological processes including neuron differentiation, mesoderm formation, palate development, cytoplasmic ribonucleoprotein granule, nucleoplasm, Golgi apparatus, cytosol, nuclear matrix, transcriptional repressor activity, RNA polymerase II transcription regulatory region sequence‐specific binding (Table 3). Three key hub genes, including *ACTB*, *SOCS1* and *ARFGEF1*, were reported in network that manifested the highest closeness (0.10, 0.10 and 0.99, respectively), betweenness (362.84, 61.43 and 105.80, respectively) and degree (10, 6 and 4, respectively) centrality. *ACTB* was identified as the most important gene related to muscle function in central of network.

**TABLE 3:** Candidate genes significantly enriched to morphometric traits‐related GO terms and the KEGG pathway.

Genes	Enrich type	Gene set	Description	–Log (*p*‐value)
*EMX2, TRAPPC9, VAX1*	BP	GO:0030182	Neuron differentiation	2.00
*POFUT2, SNAI1*		GO:0001707	Mesoderm formation	1.40
*SNAI1, VAX1*		GO:0060021	Palate development	1.10
*SOCS1, ACTB*	CC	GO:0036464	Cytoplasmic ribonucleoprotein granule	1.22
*ARFGEF1, CIITA, SOCS1, CSE1L, SNAI1, ZNF326, WARS2*		GO:0005654	Nucleoplasm	1.22
*ARFGEF1, GBP5, CLEC16A, TRAPPC9*		GO:0005794	Golgi apparatus	1.10
*ARFGEF1, CIITA, CSE1L, CLEC16A, SNAI1, NMBR, TNS3, ACTB*		GO:0005829	Cytosol	1.10
*ARFGEF1, ZNF326*		GO:0016363	Nuclear matrix	1.10
*TBX15, SNAI1, VAX1*	MF	GO:0001227	Transcriptional repressor activity, RNA polymerase II transcription regulatory region sequence‐specific binding	1.30

## 4 DISCUSSION

### 4.1 Associated SNPs

We produced over 14k markers using genotyping‐by‐sequencing (GBS) technique to carry out a genome‐wide association study (GWAS) for investigating the morphometric traits of 96 dromedaries. A total of 59 SNPs were associated with morphological measures such as height at whither, muzzle circumference, pin width, tail length, and whither to pin length, with the top significant SNPs located in Chr 11, 14 and 18. An et al. (2019) reported 24 SNPs associated with hip height, body height and body length in Chinese cattle (An et al., 2019). The nine SNPs correlated with morphometric traits in Hu sheep (Jiang et al., 2021). The top associated SNPs with chest width were identified in beef and dairy cattle (Bilal et al., 2016). The 28 chromosome‐wide significant SNP associated with body measurements and body condition score in cattle (Vanvanhossou et al., 2018). Moaeen‐ud‐Din et al. (2022) reported a number of highly significant SNPs associated with growth and body conformation traits in goat. Abdelmanova et al. (2022) identified the seven SNPs strongly associated with stature in cattle using Genome‐wide association studies.

### 4.2 Candidate genes and PPI network construction

We investigated the functions of the top associated candidate genes. *ACTB* gene codes the cardiac muscle actin, the skeletal muscle actin and the cytoplasmic beta‐actin (Czosnek et al., 1983). *DDX27* and *DEAD‐*Box RNA helicase are required for skeletal muscle growth and regeneration (Bennett et al., 2018). *ZNF326* regulates cell growth (Yu et al., 2019). Jiang et al. (2021) reported that the *ZNF521* gene is associated with body size in cross‐bred sheep. *TBX15* is required for skeletal development (Yu et al., 2019). Eight prioritised regulatory SNPs in the *TBX15/WARS2* region are risk candidates for obesity and/or osteoporosis risk (Zhang et al., 2020). *DEXI* and *CIITA* genes have effects on the immune system (Edgar et al., 2001; Mehta et al., 2011). *CLEC16A* and *ACTR3B* were associated with hip width in Simental and Angus cattle (Edgar et al., 2001). Ramírez‐Ayala et al. (2021) investigated that *PTPN1* gene can be effective in thermogenesis and hair development. Also the SNP rs3787348 in *PTPN1* was associated with the body mass index (BMI) and waist circumference among obese Japanese patients (Veyssiere et al., 2019). *TRAPPC13* and other *TRAPPC* subunits are relevant for mediating the toxicity of several small molecule compounds that induce Golgi dispersal and inhibit secretion (Ramírez‐Ayala et al., 2021). Mice with mutant *ANKRD26* were very obese and had a large body size (Bera et al., 2008). Skeletal muscle growth is regulated by *DDX27* gene through modulation of translational processes (Bennett et al., 2018). Thomas et al. (2021) reported that haploinsufficiency of *ARFGEF1* gene can cause developmental delay (Teumer et al., 2016). Also, results showed *SUPT20H* gene effects on rheumatoid arthritis (Veyssiere et al., 2019). *SNAI1* knockdown decreased the expression of genes related to cytoskeleton rearrangement and extracellular matrix (ECM) remodelling (Sun et al., 2018). Teumer et al. (2016) confirmed that *TNS3* gene associated with circulating IGF‐I and *IGFBP*‐3 concentrations. The *PGIS* gene is effective in cytoskeleton differentiation (Murray et al., 2018). Most of the candidate genes in this research correlated with growth, body size and the immune systems. Vanvanhossou et al. (2018) reported that candidate genes including *PTAFR, PBRM1, ADAM* and *TS12* associated with immune response in cattle. Bitaraf Sani et al. (2022) identified ten biological functions in dromedaries (calcium ion binding, protein binding, DNA‐binding transcription factor activity, protein kinase activity, tropomyosin binding, myosin complex, actin‐binding, ATP binding, receptor signalling pathway via JAK‐STAT, and cytokine activity) that were associated with growth (Murray et al., 2018). In this research, the lack of morphometric data and pedigrees, small herd size and missing connectedness, and genetic assessments are the main limitations.

## 5 CONCLUSION

Evaluation of morphological traits such as body length measurements of livestock are tools that can be used to predict growth rate, genetic improvement, body condition, conformation and carcass characteristics. This paper showed a genome‐wide association study of morphometric traits in dromedaries. Overall, our results revealed 59 SNPs and 35 candidate genes that can be associated with the morphometric traits. Most of them had a clear connection with growth, body size and the immune systems in other species. Interestingly, the results highlight the association between whither height (*ZNF326, GBP5, WARS2, TBX15, ZNF326, DEXI, CIITA* and *TSPYL4*), muzzle circumference (*EMX2, PRSS47, PTPN1, TRAPPC13* and *MAGEA2*), tail length (*SNAI*), and whither to pin length (*TNS3*, *TRMT9B, PTPN1* and *PTGIS*). The candidate genes are mainly associated with growth, body size and the immune system of other species. We identified three key hub genes in the PPI network including *ACTB, SOCS1* and *ARFGEF1*. In central position of gene network, *ACTB* was detected as the most important gene related to muscle function. The associated SNPs and candidate genes in this research should be validated using cell‐based systems and model organisms by like chromatin immunoprecipitation, and chromosome conformation capture methods. Afterwards, future investigations can be extended to dromedary populations from other countries to validate the findings and conclusions that can be drawn from this study.

## AUTHOR CONTRIBUTIONS

Conceptualisation, M.B.S., A.Sh.N. and J.Z.H. Methodology, M.B.S. and Z.R. Formal analysis, M.B.S. Investigation, M.B.S., N.A., A.J. and O.K. Writing – original draft preparation, M.B.S. Writing – review and editing, M.M., M.B.S., A.K. and P.A.B. Visualisation, A.J. Supervision, N.A. and P.A.B. All authors have read and agreed to the published version of the manuscript.

## FUNDING

P.A.B. acknowledges funding from the Austrian Science Fund (FWF) project P29623‐B25. This research was jointly funded by Animal Science Research Institute of Iran (ASRI), Animal Breeding Center of Iran, and Yazd Agricultural and Natural Resources Research and Education Center, grant number 34‐64‐1357‐005‐970180.

## CONFLICT OF INTEREST STATEMENT

The authors declare that the research was conducted in the absence of any commercial or financial relationships that could be construed as a potential conflict of interest.

## ETHICS STATEMENT

All procedures were performed in strict accordance with the guidelines and regulations proposed by the Animal Science Research Institute of Iran. All the animal experiments were approved by ethics committee of the Animal Science Research Institute of Iran under the number ASRI‐34−64‐1357−005‐970,180. Blood samples were collected during qualified veterinary treatment within the framework of governmental programs aimed at the animal identification, monitoring of health and parentage confirmation of the dromedary populations included in our study. No other kind of tissue was used in this study.

## Data Availability

The data sets generated and during the current study are available in the [dryad] repository, [https://datadryad.org/stash]. (Accessed on 22 April 2022).
